# U‐CIE [/juː ‘siː/]: Color encoding of high‐dimensional data

**DOI:** 10.1002/pro.4388

**Published:** 2022-08-18

**Authors:** Mikaela Koutrouli, John H. Morris, Lars Juhl Jensen

**Affiliations:** ^1^ Novo Nordisk Foundation Center for Protein Research, Faculty of Health and Medical Sciences University of Copenhagen Copenhagen Denmark; ^2^ Resource on Biocomputing, Visualization, and Informatics University of California California

**Keywords:** visualization, tool, single cell, omics, CIELAB

## Abstract

Data visualization is essential to discover patterns and anomalies in large high‐dimensional datasets. New dimensionality reduction techniques have thus been developed for visualizing omics data, in particular from single‐cell studies. However, jointly showing several types of data, for example, single‐cell expression and gene networks, remains a challenge. Here, we present ‘U‐CIE, a visualization method that encodes arbitrary high‐dimensional data as colors using a combination of dimensionality reduction and the CIELAB color space to retain the original structure to the extent possible. U‐CIE first uses UMAP to reduce high‐dimensional data to three dimensions, partially preserving distances between entities. Next, it embeds the resulting three‐dimensional representation within the CIELAB color space. This color model was designed to be perceptually uniform, meaning that the Euclidean distance between any two points should correspond to their relative perceptual difference. Therefore, the combination of UMAP and CIELAB thus results in a color encoding that captures much of the structure of the original high‐dimensional data. We illustrate its broad applicability by visualizing single‐cell data on a protein network and metagenomic data on a world map and on scatter plots.

## 1 INTRODUCTION

**FIGURE 1:**
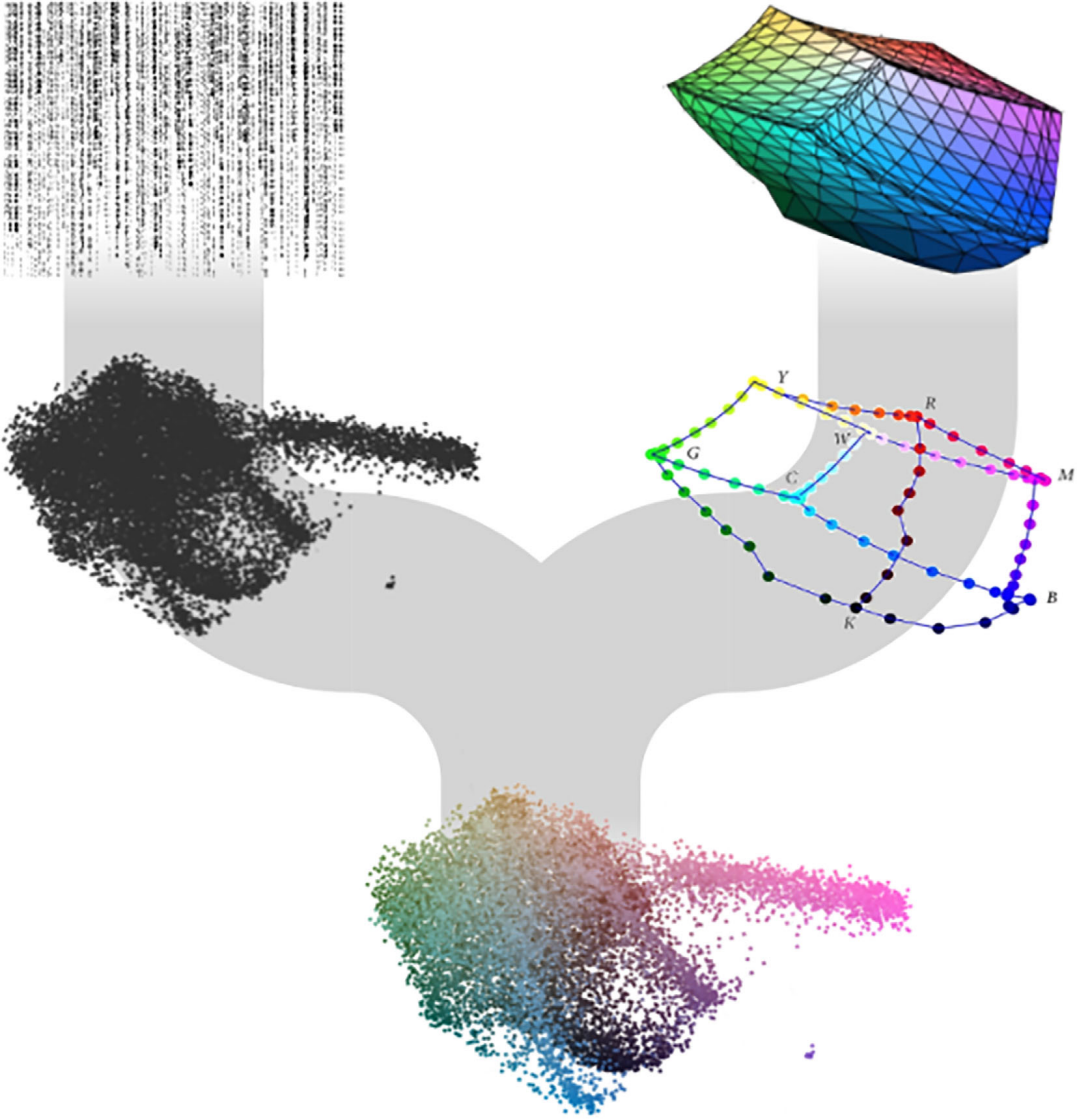
Overview of the U‐CIE algorithm and application to single‐cell RNAseq data (scRNA‐seq). U‐CIE uses a two‐step process to encode high‐dimensional data as colors. The first step is to use a state‐of‐the‐art dimensionality reduction technique (UMAP) to reduce the data to three dimensions, since human color perception is inherently three‐dimensional. The second step is to fit the resulting point cloud into the three‐dimensional polygon that represents the displayable part of CIELAB color space. This is done as an optimization process, which shifts, rotates, and uniformly scales the point cloud to make it as large as possible while penalizing for points protruding outside the polygon

Today large, high‐dimensional datasets are abundant in biomedicine. Data visualization is thus crucial both for discovering patterns in data and for subsequently communicating the insights. With this motivation, we present U‐CIE [/juː ‘siː/] an open‐source software tool that translates high‐dimensional data into colors. As color space is inherently three‐dimensional, U‐CIE does this in two steps: a three‐dimensional approximation of the high‐dimensional data is produced using a dimensionality reduction method, and this approximation is next fitted into a suitable color space (Figure 1). Each high‐dimensional input vector, be it the expression profile of a gene or the abundance profile of an organism, is thereby converted into a color. These colors can subsequently be used to visualize the, for example, genes or organisms in the context of other data, such as gene networks, phylogenetic trees, or longitude and latitude.

Dimensionality reduction techniques have improved much in recent years, becoming better at preserving more of both the local and global structures in high‐dimensional data by making nonlinear rather than linear transformations.^1^ Methods including t‐SNE^2^ and UMAP^3^ as well as generative deep‐learning models, such as variational autoencoders,^4^ have become particularly popular within biology, especially for visualization of single‐cell data. Both t‐SNE and UMAP were designed to predominantly preserve local structure by grouping neighboring data points together, which provides a very informative visualization.^2^, ^3^ The main difference between t‐SNE and UMAP is the interpretation of the distance between clusters. UMAP preserves pairwise Euclidean distances significantly better than t‐SNE, meaning that it preserves more of the global structure along with the local. Therefore, the relative positioning of different clusters in t‐SNE is not informative about the distance between them, which is why UMAP has gained popularity for visualization of single‐cells studies. An alternative is to use a variational autoencoder (VAE), which is an artificial neural network that is trained to compress the data to a lower dimensional representation from which the input can be reconstructed.^4^ Even though VAEs can be more accurate in the low‐dimensional representation, they are not commonly used yet, since they are more difficult to use than t‐SNE and UMAP.

Colors are everywhere and we all have an intuitive understanding of colors; however, it is surprisingly difficult to represent them in a way that accurately reflects how similar humans perceive any two colors to be. None of the most commonly used color representations, such as RGB, approximate human vision. A notable exception is the CIELAB color model,^5^ which was designed to be largely perceptually uniform, meaning that the Euclidean distance between any two points should correspond to their relative perceptual difference. This makes it particularly useful for our application, since we want to visualize positions as colors, similar to what has previously been tried for self‐organizing maps.^6^ Indeed, experiments have shown that users are able to identify clusters in high‐dimensional data when encoded in the CIELAB color space.^7^ CIELAB represents colors using three values: perceptual lightness (L*) and two pairs of complementary colors, namely green–red (a*) and blue–yellow (b*). To take advantage of the properties of CIELAB, we need to convert the three‐dimensional coordinates obtained from dimensionality reduction into L*a*b* coordinates. This is nontrivial, because not all combinations of L*, a*, and b* result in colors that can be displayed on a computer monitor.

Here, we present the U‐CIE visualization method for encoding high‐dimensional data as colors. We first use UMAP to turn the data into a three‐dimensional point cloud, as UMAP partially preserves global Euclidean distances. Next, we use an optimization algorithm to fit the point cloud within the displayable part of CIELAB. The result is an encoding of the input data, where each data point has been assigned a color, and similarity between colors reflects the distance between the original points. We illustrate the broad applicability of U‐CIE by using it to visualize single‐cell expression data on a protein network and microbiome composition of ocean water samples on a world map. U‐CIE is freely available both as a web resource (https://u-cie.jensenlab.org) and as an R package.

## 2 MATERIAL AND METHODS

### 2.1 Algorithm overview

U‐CIE color encodes high‐dimensional data using CIELAB color space in two stages: (i) dimensionality reduction (can be skipped if data are already three‐dimensional) and (ii) fitting the resulting point cloud inside the displayable part of CIELAB (Figure 1). The latter is a precomputed polygon, constructed by converting the RGB cube to CIELAB coordinates.

### 2.2 Dimensionality reduction

Unless the input data are already three‐dimensional, U‐CIE will start by reducing the data to three dimensions. There are three different tracks for doing so: “Single cells”, “High dimensional data”, and “Distance matrix”. The ‘Single cells’ track follows the Seurat^8^ pipeline to reduce dimensionality and produce 3D UMAP coordinates for the genes of the dataset. To do so, we first transpose the input dataset to have genes as columns and cells as rows and create a Seurat Object from the counts. Without first scaling or centering the data, we use Seurat to log2 transform our data using the (option “LogNormalize”), run PCA with 50 dimensions, calculate 50 neighbors for each gene, and find clusters with the Louvain algorithm. Finally, we apply UMAP to end up with three dimensions. The “High dimensional” track uses the uwot library to again run PCA with 50 dimensions and apply UMAP. Finally, the “Distance matrix” track takes a square matrix and uses Python umap‐learn package.

### 2.3 Fitting the point cloud inside the CIELAB RGB polygon

To be able to handle large datasets with many points, we first use the R “chull” function to construct the convex hull of the point cloud. This dramatically expidites the subsequent optimization step, typically by several orders of magnitude, as it only has to consider the few points on the convex hull rather than all points in the input dataset. Next, we use the Nelder–Mead simplex optimization algorithm^9^ to fit the convex hull of the point cloud inside the CIELAB RGB polygon. The algorithm is allowed to shift along and rotate around three axes (L*, a*, and b*) and uniformly scale the point cloud convex hull. The objective function to be optimized is the size of the point cloud minus a penalty term for points falling outside the polygon. In other words, it aims to make the point cloud as large as possible while still fitting within the color space. To avoid local optima, we run the algorithm with 25 different sets of initial rotations. The user can optionally provide different weights for the axes in the objective, thus prioritizing spreading out the points along certain axes.

### 2.4 User interface

The web interface was constructed using R/Shiny and JavaScript. An interactive guided tutorial is available through the web interface as well as a YouTube video explaining the idea of U‐CIE.

After uploading and processing data via one of the tracks described above, the data can be visualized in two ways. The “3D view” shows the main result of following the U‐CIE pipeline, namely the 3D cloud of points colored according to the best solution found by the optimization algorithm. The view also has a table of alternative color solutions from the optimizations, which can be selected and displayed instead. The “2D projections” tab provides two interactive 2D plots, showing the same 3D point cloud projected onto the L*a* and L*b* axes, respectively. There users can see how the points are spread within the polygon. The view also has a table, which allows the user to select regions within the plots to identify the data points or search the data points by name to locate them within the cloud. Finally, the “Download” tab shows a table with both RGB hex codes and CIELAB coordinates, which can be downloaded to use the colors for further data visualization, for example, in Cytoscape [8]. The plots and tables are made using the Plotly^10^ and DataTables CRAN.R-project.org/package=data.table libraries, respectively.

### 2.5 CRAN package “ucie”

Our algorithm is also available as a CRAN package in R. Users can download it from R with the command install.packages(“ucie”) or from the packages and the command devtools::install_github(“mikelkou/ucie”). The package returns a data frame with the names of the input data points and RGB hex colors or CIELAB coordinates. The package contains three functions, one which does the optimization, one that does the color production, and one that does both steps. For the optimization, users must provide a three‐column dataset that will be encoded as colors and can optionally alter the axis weights. The output is an array with the parameters for the optimal transformation into CIELAB coordinates. For the color production, the user must provide the three‐column dataset, the transformation parameters, optionally a final scaling factor, and whether they want RGB hex codes or CIELAB coordinates. The output is a data frame with the names of the input data points and the corresponding colors.

## 3 RESULTS AND DISCUSSION

### 3.1 U‐CIE [/juː ‘siː/] an open‐source software tool

To allow anyone to easily use U‐CIE, we have made it available as an interactive web resource (https://u-cie.jensenlab.org) along with a guided tutorial. The web interface offers four different tracks: “Single cells”, “High dimensional”, “Distance matrix”, and “3D data”. All use UMAP to first reduce dimensionality, except from the last track, which allows the user to upload data that is already three‐dimensional, possibly produced using another dimensionality reduction method. In Figure S1 we show how an analysis can be performed using the web interface. First, we upload the matrix with expression counts to the “Single cells” track, which assigns colors to genes. A preview of the uploaded data is first shown, allowing us to verify that it was parsed correctly before starting the analysis. As soon as the analysis is finished, we gain access to interactive visualization panels that show the color encoding in 3D and as 2D projections, controls to alter the color encoding, and the option to download a tab‐delimited file with the gene names and their colors.

U‐CIE is also available as an R package. Since users of this package will be working in the R environment, we give them full flexibility to use any dimensionality reduction method for creating the three‐dimensional representation of the input data. The R package thus takes this as input and performs the optimization to fit the data into CIELAB coordinates. The output is a data frame with the names of the input data and the hex codes of the colors or the CIELAB coordinates. This data frame can be directly used with R or saved as a file locally for use in other visualization tools. Both the R package and the web resource are available under the open source MIT License.

### 3.2 scRNA‐seq data on physical protein complexes

**FIGURE 2:**
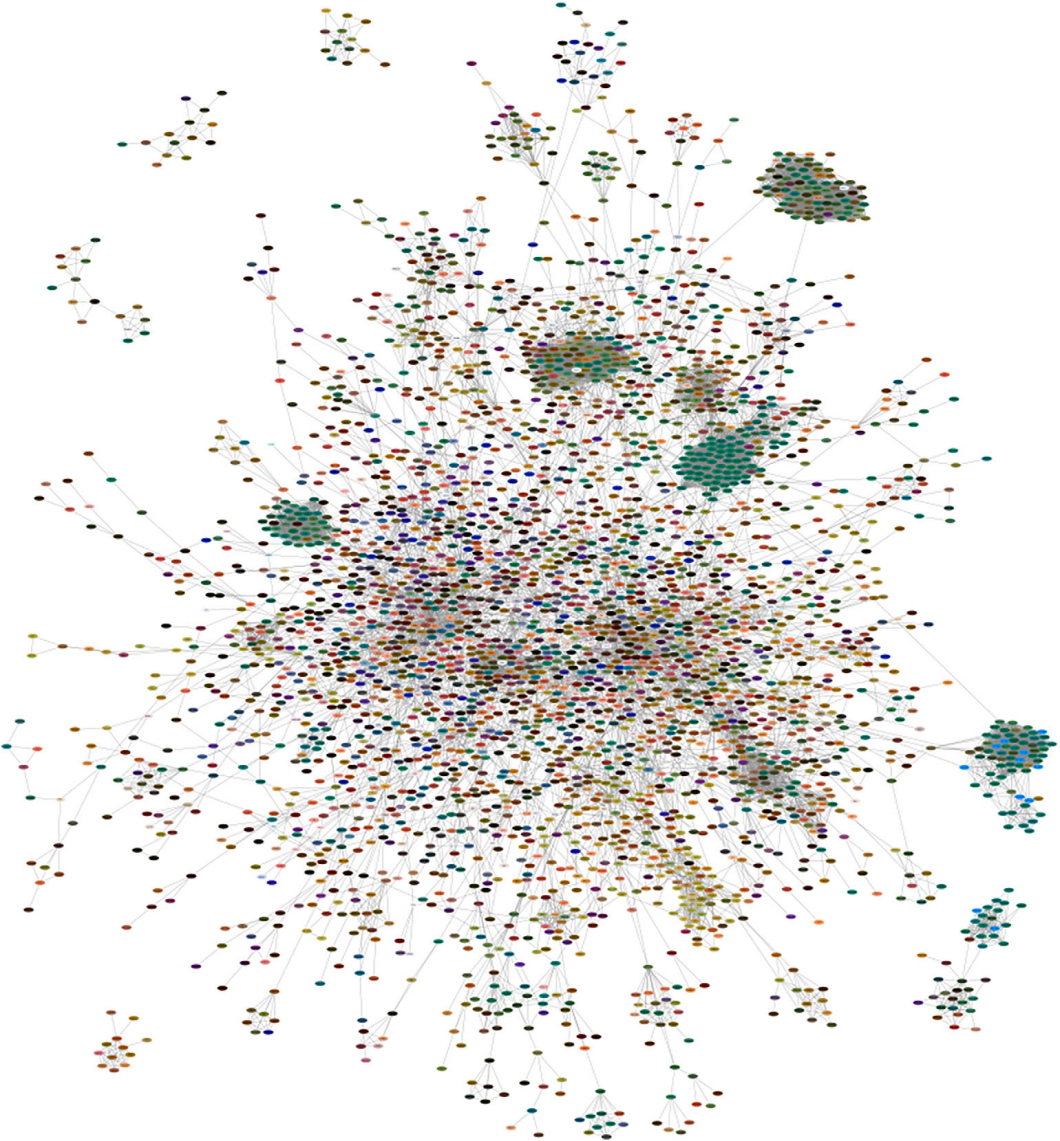
Color encoding the scRNA‐seq on physical protein complexes. To illustrate how U‐CIE can be used to visualize expression data on protein networks, we downloaded a published scRNA‐seq dataset with a read‐count matrix of 1,018 cells.^14^ We applied U‐CIE to the transposed matrix (genes as columns, cells as rows), exported the colors, and visualized them on a physical protein–protein interaction network from the STRING database^7^ (confidence cutoff 0.95) using Cytoscape.^8^ In the figure, we show the six largest connected components. The network contains several large dark green clusters; these correspond to large complexes of housekeeping proteins, such as the cytosolic and mitochondrial ribosomes, the proteasome, and the electron transport chain complexes. The latter also contain some blue subunits, which are the proteins encoded by the mitochondrial genome. The less obvious cluster of tan nodes corresponds to core cell‐cycle proteins, including cyclin‐dependent protein kinases and the DNA replication complexes

Having a color encoding of high‐dimensional data is useful whenever a user wants to visualize the data in the context of some other information, since it frees up the spatial coordinates. For example, transcriptomics and proteomics data are commonly visualized onto gene/protein networks. We exemplify this use of the U‐CIE method by applying it to network visualization of a scRNA‐seq data of 19,097 genes across 1,018 cells. We used U‐CIE to convert these data to a color for each gene and mapped them on a physical protein interaction network from STRING v11.5^11^, ^12^ (confidence cutoff 0.95) using Cytoscape.^13^ This allows us to graphically summarize the transcriptional regulation of the individual subunits within protein complexes. Figure 2 shows the six largest connected components of the network. Several clusters corresponding to large protein complexes with many green subunits stand out in the network, including the cytosolic and mitochondrial ribosomes, the proteasome, and the electron transfer chain complexes. As these carry out housekeeping functions it makes sense that they have very similar expression patterns across cells and thus have the same color. The electron transfer chain complexes also have a few blue subunits, which are the ones encoded by the mitochondrial chromosome. The network also contains a cluster of tan proteins, which are involved in core cell‐cycle processes, such as the cyclin‐dependent protein kinases and DNA replication complexes. This example illustrates that U‐CIE is able to assign colors to genes based on scRNA‐seq data in a biologically meaningful way.

### 3.3 Microbiome compositions on a world map

**FIGURE 3:**
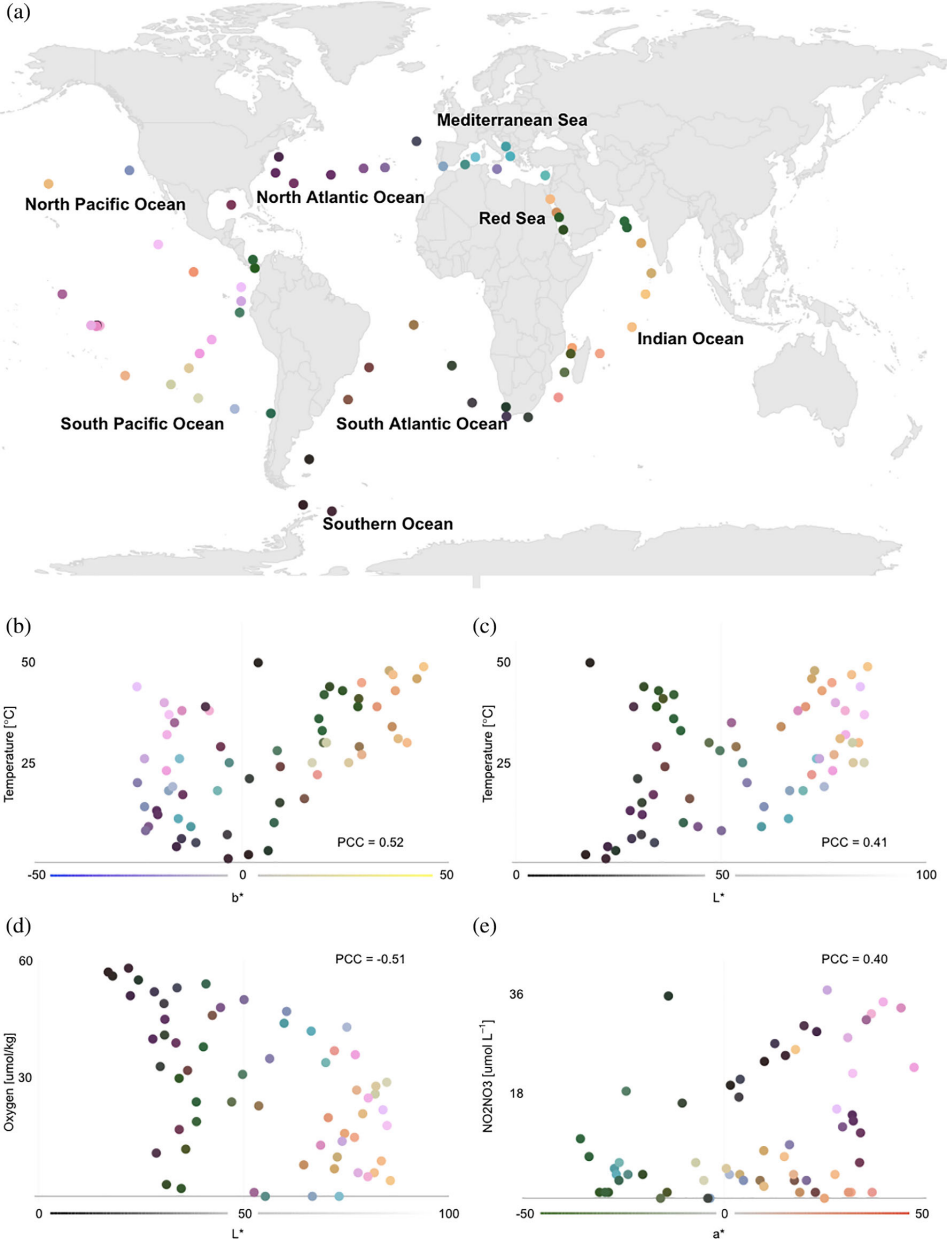
Color encoding the microbiome compositions of ocean surface water samples. 16S rRNA microbiome composition of surface water samples from the Tara Oceans^15^ sampling stations were encoded as colors using U‐CIE. The resulting colors were mapped onto the geographic locations of the sampling stations (panel a). The blue–yellow (b*) axis correlates with water temperature (panel b), the lightness (L*) correlates positively with temperature dissolved (panel c) and negatively with oxygen concentration (panel d), and the green–red axis (a*) correlates with dissolved nitrite/nitrate concentration (panel e)

U‐CIE is not limited to single‐cell data. Figure 3 shows a completely different use case, namely visualization of microbiome compositions. Specifically, we used U‐CIE to color the sampling stations of the Tara Oceans project^15^ based on their 16S taxonomic profiles from surface water samples. Combining these colors with the longitudes and latitudes of the sampling stations enables us to show the microbiome compositions on a world map (Figure 3a). This makes it immediately clear that the samples from some oceans have quite similar microbiome composition. For example, samples from the Mediterranean Sea tend to be blue while samples from the Indian Ocean tend to be yellow (Figure 3a). The color coding of the samples on the map thus allows users to easily see which samples cluster in terms of having similar microbiome compositions.

In addition to allowing clusters to be visually identified, the colors produced by U‐CIE can also be interpreted in terms of environmental factors that drive microbiome composition. We did this by correlating each factor measured in the Tara Oceans project with the individual CIELAB color axes. The strongest correlation found is that the blue–yellow axis (b*) correlates with water temperature (PCC = 0.52; Figure 3b). This agrees with the observation in the original publication that temperature correlates strongly with microbiome composition.^15^ The lightness (L*) also correlates with water temperature (PCC = 0.41; Figure 3c) but shows a stronger negative correlation with the dissolved oxygen concentration (PCC = ‐0.51; Figure 3d). Together, these axes thus correctly capture, from 16S abundance profiles, that the Indian ocean is warm with low oxygen, the North Atlantic Ocean is cold with high oxygen, and the Pacific Ocean has low oxygen and highly varying temperatures. Finally, the green–red axis (a*) correlates with dissolved nitrite/nitrate concentration (panel E); for example, the middle of the Pacific Ocean is a high‐nitrogen environment, whereas the waters around Panama have low nitrogen.

### 3.4 User‐provided dimensionality reduction

The two examples illustrate two of the three tracks in the U‐CIE web resource, which use UMAP to compress high‐dimensional data into three dimensions and subsequently convert it to colors. However, dimensionality reduction is a hard task that inherently involves distorting distances, and this can result in different data points in the original high‐dimensional space mapping to the same color. Moreover, UMAP will not be the best choice of algorithm for all datasets.

For this reason, U‐CIE has a fourth track that allows users to directly upload 3D data. While this obviously allows data that are inherently 3D to be converted to colors, the main purpose is to allow users to apply any dimensionality reduction algorithm to their data before providing it to U‐CIE for conversion into colors. This more closely mimics how the R package works and furthermore ensures a one‐to‐one mapping between the user‐provided data and colors. The optimal parameters for the CIELAB color conversion can be applied to new data points through the R package.

## 4 SUMMARY

We have developed a new visualization tool, U‐CIE, which allows arbitrary high‐dimensional data to be encoded as colors. The method first uses existing dimensionality reduction techniques, for example, UMAP, to reduce the input data to three dimensions, and next embeds this representation of the data within the CIELAB color space. We illustrate the usefulness of U‐CIE by applying it to (i) visualization of scRNA‐seq data on a physical protein interaction network and (ii) visualization of microbiome composition from sampling stations on a world map. U‐CIE is available both as a web resource at https://u-cie.jensenlab.org/ and as an R package.

## AUTHOR CONTRIBUTIONS

**Mikaela Koutrouli:** Formal analysis (lead); methodology (lead); software (lead); visualization (lead); writing – original draft (lead); writing – review and editing (lead). **John H. Morris:** Conceptualization (equal); supervision (supporting). **Lars Juhl Jensen:** Conceptualization (lead); supervision (lead); writing – original draft (supporting); writing – review and editing (equal).

## FUNDING INFORMATION

This work was supported by the Novo Nordisk Foundation [NNF14CC0001] and [NNF20SA0035590].

## CONFLICT OF INTEREST

The authors declare no conflicts of interest.

## Supporting information


**FIGURE S1** Web‐resource interface of U‐CIE. The web interface was constructed using R/Shiny and JavaScript. The first choice users need to make is which track is more suitable for their data. After uploading and processing data via one of the tracks, there are two outputs of the data. The “3D view” shows the best solution found by the optimization algorithm following the U‐CIE pipeline, namely the 3D representation of the cloud of points colored. Alternative colors can be selected and displayed instead from the table of alternative color solutions from the optimizations. The “2D projections” tab provides two interactive 2D plots, showing the same 3D point cloud projected onto the L*a* and L*b* axes, respectively. There users can see how the points are spread within the polygon. Finally, from the “Download” tab users can download the colors and use them for further data visualization, for example, in Cytoscape. The plots and tables are made using the Plotly and DataTables libraries, respectively.

## Data Availability

Each track offers a different small example dataset. The “Single cells” track uses a single‐cell RNA‐seq dataset of human embryonic stem cells (GEO accession code GSE75748).^14^ The “High dimensional” track uses an Affymetrix time course dataset of *Drosophila melanogaster* embryogenesis.^16^ The “Distance matrix” example uses the bacterial species tree available from the iTOL web resource (https://itol.embl.de).^17^ For the “3D Data” track we used the same dataset as in the ‘High dimensional’ track, reduced to three dimensions with locally linear embedding. Finally, the dataset used to create Figure 2 comes from^15^ and can be downloaded from http://ocean-microbiome.embl.de. The U‐CIE source code is available at https://github.com/mikelkou/U-CIE_Web_Resource. The R package “ucie” code is available at https://github.com/mikelkou/ucie, through CRAN (install.packages(“ucie”)), and through devtools (devtools install_github(“mikelkou/ucie”)).
